# SCAMP2/5 as diagnostic and prognostic markers for acute myeloid leukemia

**DOI:** 10.1038/s41598-021-96440-2

**Published:** 2021-08-23

**Authors:** Can Yue, Siting Xie, Jiaying Zhong, Haijun Zhao, Zhijuan Lin, Li Zhang, Bing Xu, Yiming Luo

**Affiliations:** 1 Graduate College of Fujian Medical University, Fuzhou, 350108 Fujian People’s Republic of China; 2 Department of Hematology, The First Affiliated Hospital of Xiamen University, Teaching Hospital of Fujian Medical University, No. 55 ZhenHai Road, Si Ming District, Xiamen, 361003 Fujian People’s Republic of China; 3 Organ Transplantation Institute of Xiamen University, Fujian Provincial Key Laboratory of Organ and Tissue Regeneration, School of Medicine, Xiamen University, Xiamen, 361003 Fujian People’s Republic of China

**Keywords:** Cancer, Biomarkers

## Abstract

The secretory carrier-associated membrane proteins (SCAMPs) are associated with the development of multiple human cancers. The role of SCAMPs in acute myeloid leukemia (AML), however, remains to be identified. In the present study, we explored expression patterns and prognostic value of SCAMPs and network analysis of SCAMPs-related signaling pathways in AML using Oncomine, GEPIA, cBioPortal, LinkedOmics, DAVID and Metascape databases. Genetic alteration analysis revealed that the mutation rate of SCAMP genes was below 1% (9/1272) in AML, and there was no significant correlation between SCAMPs gene mutation and AML prognosis. However, the SCAMP2/5 mRNA levels were significantly higher in AML patients than in healthy controls. Moreover, high mRNA expressions of SCAMP2/4/5 were associated with poor overall survival, which might be due to that SCAMP2/4/5 and their co-expressed genes were associated with multiple pathways related to tumorigenesis and progression, including human T-cell leukemia virus 1 infection, acute myeloid leukemia, mTOR and NF-kappa B signaling pathways. These results suggest that SCAMP2/4/5 are potential prognostic markers for AML, and that SCAMP2 and SCAMP5 individually or in combination may be used as diagnostic markers for AML.

## Introduction

The secretory carrier-associated membrane proteins (SCAMPs), a family of transcription factors encoded by five SCAMP genes in eukaryotes, are ubiquitously expressed in secretory membrane^1^. SCAMPs control intracellular trafficking and signaling involved in cell–cell adhesion, cancer migration and invasion^2–6^. Based on the variable presence of multiple N-terminal asparagine-proline-phenylalanine (NPF) repeats, human SCAMPs are divided into two groups: SCAMP1/2/3 (with NPF repeats) and SCAMP4/5 (without NPF repeats), whereas, SCAMPs with the same NPF repeats may have distinct functions. Both groups have four central transmembrane regions (TMRs) and cytoplasmic tail. TMRs participate in membrane transport and traffic with their interacting partners on the membrane-cytosol interface^1,7,8^. Importantly, alterations in cytoskeletal pathways mediated by SCAMPs can affect cell–cell adhesion and may result in cell polarity loss and the increase of cell motility and invasion via changing components of the plasma membrane^5,8–10^. In consistent with these reports, dysregulation of SCAMPs has been found in series of human malignancies, such as hepatocellular carcinoma^11^, lung cancer^12^; breast cancer^5^, colorectal cancer^2^, ovarian cancer^3^, pancreatic cancer and gallbladder cancer^6,13^. Recently, the role of SCAMPs in human cancers has been a topic of increasing interest.

Acute myeloid leukemia (AML) is a malignant clonal disease of hematopoietic tissue characterized by dysregulated proliferation, impaired apoptosis, and disrupted blasts of myeloid lineage differentiation, accompanied by severe infections, anemia and haemorrhage^14,15^. AML is the most common subtype in adult acute leukemia, accounting for 80% morbidity. It is estimated that there will be 20,240 new cases, and 11,400 deaths from AML in 2021 in USA^16^. Despite improvements in multi-agent chemotherapy, chemoimmunotherapy, targeted therapy and allogeneic stem cell transplantation for clinical management of leukemia, the 5-year relative survival was only 29.5% (2011–2017) due to chemotherapy resistance, immune rejection, and poor adherence to treatment^16–20^. Notably, current medical science lacks reliable and efficient prognostic biomarkers to enable early diagnosis and accurate prediction of prognosis for AML. Therefore, there is an urgent need to explore molecular biomarkers and therapeutic targets to enhance prognostic capabilities and to promote individualized treatment in the era of precision medicine.

Up to this point in time, the roles of SCAMPs in AML remain poorly understood. Herein, we comprehensively analyzed the relationships between the five SCAMPs and AML based on the data from Oncomine, GEPIA (Gene Expression Profiling Interactive Analysis), cBioPortal, LinkedOmics and DAVID (the Database for Annotation, Visualization and Integrated Discovery), as a means of assessing SCAMP expression patterns, potential functions, and prognostic utility in the context of AML.

## Materials and methods

### Ethics statement

This study has been approved by the Ethics Committee of the First Affiliated Hospital of Xiamen University (Fujian, China). The data was retrieved from published literature, and all analysis were performed in accordance with the Declaration of Helsinki.

### Oncomine database analysis

The Oncomine v4.5 (https://www.oncomine.org) database is an online tool to analyze, standardize, and process tumor microarray transcriptomic data. This database was used to analyze SCAMP expression profiles between AML samples and normal controls. The normal controls were data from bone marrow. P-values were generated through Student’s t-tests, and the cut-off criteria for Oncomine analyses in this study were a p-value of 0.01 and a fold-change value of 2^21^**.**

### GEPIA analyses

The GEPIA (http://gepia.cancer-pku.cn) platform can be used to analyze differential expression profiles associated with various types of tumors in The Cancer Genome Atlas (TCGA) (http://tcga-data.nci.nih.gov/tcga/) and the Genotype-Tissue Expression Project (GTEx) (http://www.gtexportal.org/home/index.html) databases, incorporating RNA-seq expression data for 9,736 tumor samples and 8,587 control samples. Here GEPIA was used for distinct SCAMP isoforms expression in AML, and it was also used to observe the relationship between SCAMP expression and overall survival of patients with AML. Data from healthy whole blood samples were used as normal controls^22^.

### LinkedOmics dataset

LinkedOmics (http://www.linkedomics.org/admin.php) is a software tool used to disseminate data pertaining to all 32 cancer types included in the TCGA with a focus on interpreting and discovering attributes associations, thereby complementing other available databases. This database allows for analyses of multiomic datasets^23^. We conducted a co-expression analysis for SCAMPs using the LinkedOmics AML datasets.

### cBioPortal dataset analyses

CBioPortal (http://cbioportal.org) leverages the GEO (Gene Expression Omnibus) and TCGA databases, profiling mutations, copy-number alterations (CNAs) from GISTIC (Genomic Identification of Significant Targets in Cancer), and mRNA and protein expression Z-scores (RNASeq V2 RSEM and RPPA)^24,25^. Alteration of SCAMP genes status in AML patients was determined using the online cancer genomics cBioPortal.

### Functional enrichment analysis

The DAVID v6.8 (https://david.ncifcrf.gov/) database^26,27^ was utilized to conduct Kyoto Encyclopedia of Genes and Genomes (KEGG) and gene ontology (GO) analyses of SCAMPs. Moreover, GO enrichment analysis were used to assess putative biological processes (BP), molecular functions (MF), and cellular components (CC) associated with genes of interest. KEGG analysis defined the pathways associated with SCAMP2/4/5 functions and their co-expression genes. Analyses utilized the human genome as a background parameter. *P* < 0.05 was the significance threshold.

### Metascape analysis

Metascape (http://metascape.org) was employed for process and pathway enrichment analyses of genes co-expressing with SCAMP2/4/5 in leukemia. As an effective, efficient and user-friendly gene-list analysis tool, Metascape enables multi-platform analyses of multi-omic data^28^. Only terms meeting the following criteria were deemed significant: *p* < 0.01, minimum count 3, and enrichment factor > 1.5. The core protein–protein interaction (PPI) network was constructed by BioGRID, InWeb_IM and OmniPath, and the results were visualized with Metascape. Then MCODE (Minimal Common Oncology Data Elements) cluster analysis was performed to detect key MCODE clusters via setting parameters to the most central and densely connected clusters in the PPI network, those being degree cutoff = 2, node score cutoff = 0.2, K-score = 2 and MAX depth = 100. Further, the functions of the most significant modules chosen from the PPI network were predicted using Metascape at a significance of *p* < 0.05.

## Results

### Genetic alteration of SCAMP genes in AML

**Figure 1:**
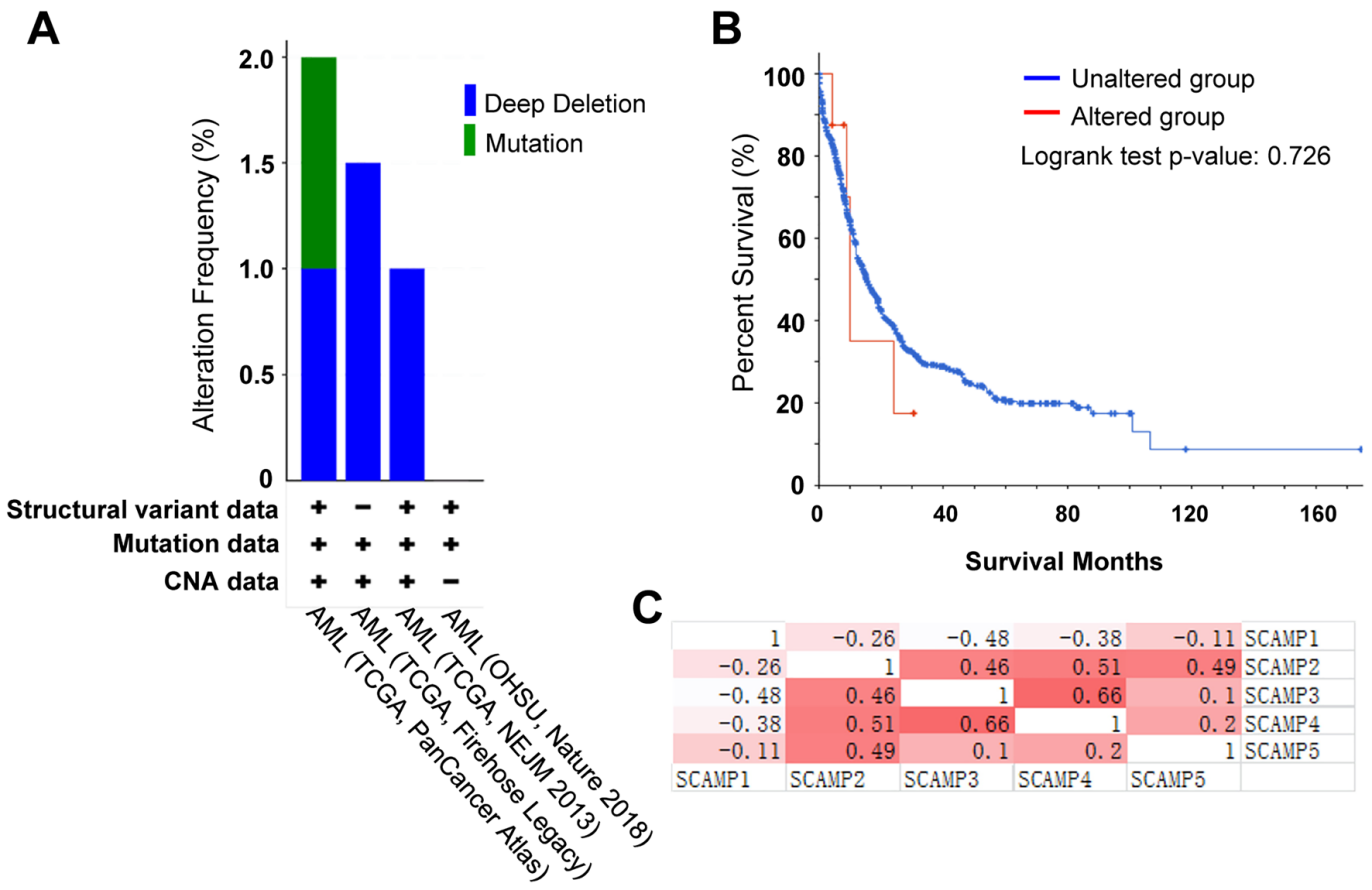
Alteration frequency of SCAMP genes has no significant correlation with AML prognosis (TCGA and cBioPortal). (**A**) OncoPrint visualization of alterations associated with SCAMPs genes. (**B**) Survival percentage and survival time of AML patients with/without gene alterations by Kaplan–Meier plots. (**C**) Correlation between the mRNA expression of different SCAMPs in AML (cBioPortal).

Genetic alteration represents one of the main causes to cancer. To investigate the correlation between SCAMPs genetic alteration with AML, we used the cBioPortal online tool to collect data of a total of 1272 samples from the TCGA provisional dataset of AML for analysis. SCAMP genetic changes were evident in 9 samples from 1162 AML patients; that is to say, the total alteration rate is below 1%. These genetic changes include deep deletion (a homozygous deletion) and mutation (missense mutation and truncating mutation) (Fig. 1A). The genetic alteration rate of SCAMP1, SCAMP2, SCAMP3, SCAMP4 and SCAMP5 was 0.2%, 0.3%, 0%, 0.4% and 0.3%, respectively. Based on the TCGA provisional dataset, Kaplan–Meier plots were used to evaluate the relationship between SCAMP family gene alteration and overall survival of cases. The result showed no significant difference in the overall survival between SCAMPs gene altered group and unaltered group (Fig. 1B).

In addition, we calculated the correlations of SCAMPs with each other by analyzing their mRNA expressions via the cBioPortal online tool for AML (The Cancer Genome Atlas, Provisional), with Pearson’s correction involved. The results demonstrated significant and positive correlations between SCAMP genes expression (Fig. 1C).

### SCAMPs expression in AML

**Figure 2:**
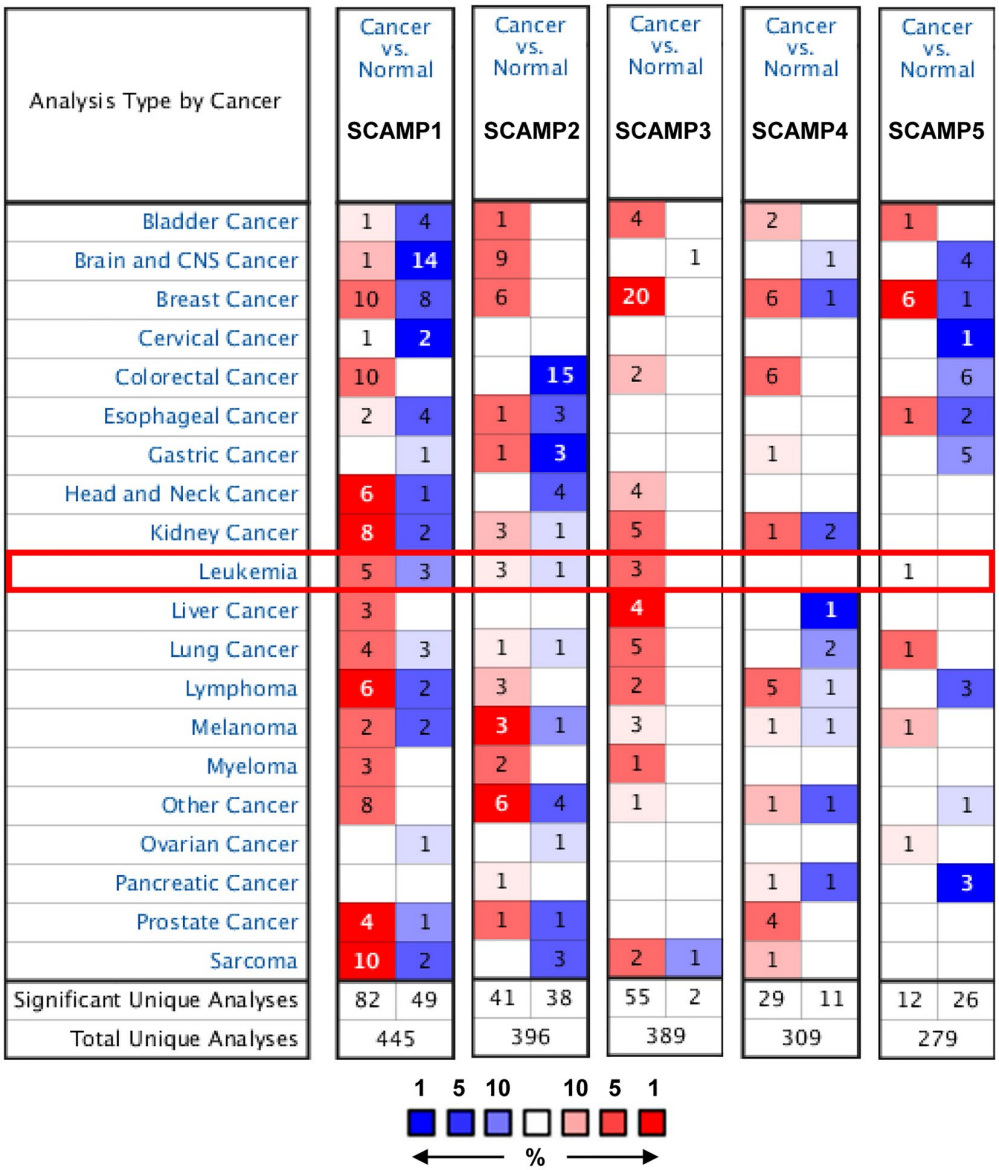
SCAMP mRNA expression in a range of cancers and leukemia (ONCOMINE database). Threshold values: *P* value = 0.001; fold-change = 1.5.

We then focused on the relationship between mRNA expression of SCAMPs and leukemia. We used the Oncomine database to compare the expression of SCAMPs in tumor samples and normal controls. As shown in Fig. 2 and Table 1, the mRNA level of SCAMP1 was significantly upregulated in leukemia patients from five datasets: childhood acute lymphoblastic leukemia (ALL) datasets from Coustan-Smith’s dataset^29^, acute adult T-cell leukemia (ATL) datasets from Choi’s dataset^30^, and AML datasets, T-cell Acute Lymphoblastic Leukemia (T-ALL) datasets and B-cell Acute Lymphoblastic Leukemia (B-ALL) datasets from Andersson’s dataset^31^. SCAMP1 was overexpressed in childhood ALL (fold change (FC) = 1.727) from Coustan-Smith’s dataset, and in acute ATL (FC = 1.505) relative to normal controls from Choi’s dataset. The expression of SCAMP1 was significantly higher in AML (FC = 1.786), T-ALL (FC = 1.537), and B-ALL patients (FC = 1.583) from Andersson’s dataset (Table 1). Similarly, the transcriptional level of SCAMP2 was significantly upregulated in patients with leukemia in three datasets (Fig. 2, Table 1). In Andersson’s dataset, the transcriptional levels of SCAMP2 were found elevated in AML (FC = 2.219), in T-ALL (FC = 2.223), and in B-ALL patients (FC = 2.170). A similar trend was also found for SCAMP3 and SCAMP5. SCAMP3 showed higher expression in AML with a fold change of 2.988, in T-ALL with a fold change of 2.016 and in B-ALL with a fold change of 2.227 compared to normal controls in Andersson’s dataset; and SCAMP5 showed overexpression in AML with a fold change of 1.725. No transcriptional expression data of SCAMP4 was found in the Oncomine database. Interestingly, the mRNA levels of SCAMP 1/2/3/5 were significantly higher in AML patients compared to normal controls in Andersson’s dataset (Table 1).

**Table 1:** The mRNA levels of SCAMPs in different types of leukemia (ONCOMINE).

Family	Types of cancer vs. normal	t-test	Fold change	*P* value	Ref
SCAMP1	Childhood ALLvs. Normal	10.162	1.727	**1.69E-8**	Smith Leukemia
	Acute ATL vs. Normal	8.753	1.505	**2.00E-03**	Choi Leukemia
	**AML vs. Normal**	4.981	1.786	**3.96E-4**	Andersson Leukemia
	T-ALL vs. Normal	2.911	1.537	**7.00E-3**	Andersson Leukemia
	B-ALL vs. Normal	4.205	1.583	**2.00E-3**	Andersson Leukemia
SCAMP2	**AML vs. Normal**	4.339	2.219	**1.00E-3**	Andersson Leukemia
	T-ALL vs. Normal	3.804	2.223	**2.00E-3**	Andersson Leukemia
	B-ALL vs. Normal	4.762	2.170	**2.00E-3**	Andersson Leukemia
SCAMP3	**AML vs. Normal**	10.705	2.988	**1.59E-8**	Andersson Leukemia
	T-ALL vs. Normal	7.517	2.016	**9.83E-7**	Andersson Leukemia
	B-ALL vs. Normal	11.751	2.2270	**2.45E-7**	Andersson Leukemia
SCAMP4	n.i				
SCAMP5	**AML vs. Normal**	2.978	1.725	**4.00E-03**	Andersson Leukemia

Bold values in the table represent statistical significance. (Threshold *P*-value < 0.05; fold change ≥ 1.5 and gene rank: all). n.i., no information.

### Prognostic values of SCAMP family members in AML

**Figure 3:**
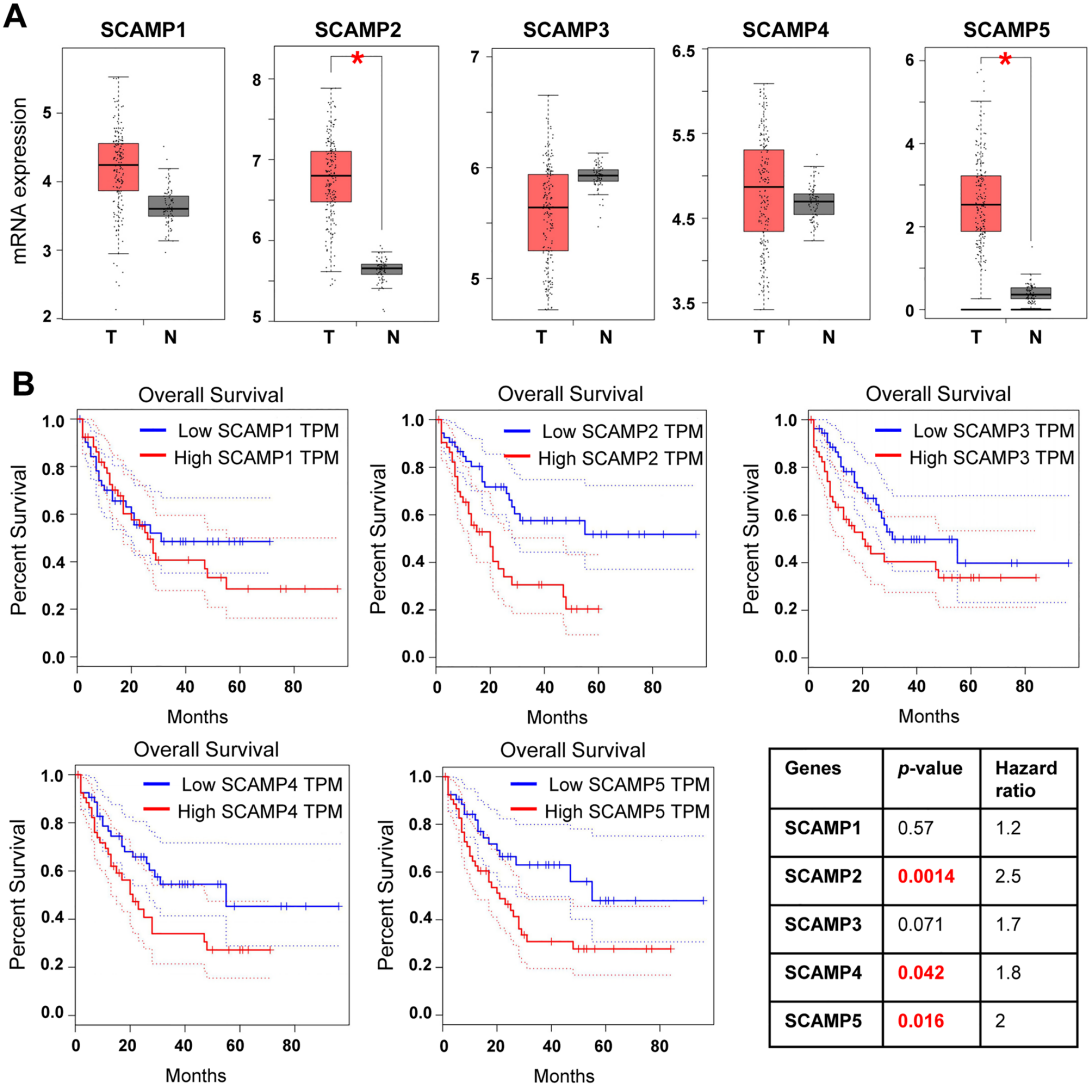
SCAMP mRNA levels and the prognostic value of the individual SCAMP in AML (GEPIA database). (A) The distribution of SCAMP1-5 gene mRNA expression between AML and normal controls. n (tumor, T) = 173; n (normal control, N) = 70. (B) Curves show relative expression of SCAMP1-5 with overall survival between AML and normal controls using GEPIA. For SCAMP1, n (high) = 52, n (low) = 52. For SCAMP2-5, n (high) = 53, n (low) = 53. *P* value of log-rank and hazard ratio were listed.

We used GEPIA to compare the correlation between SCAMPs expression level and patient survival rate. The mRNA levels of SCAMP2 and SCAMP5 showed significantly higher in AML patients than in normal controls (Fig. 3A). Interestingly, the overall survival rates related to these two SCAMPs also showed significant differences between cases with low SCAMPs level and cases with high SCAMPs level through GEPIA curve and log-rank test analyses (Fig. 3B). Although there wasn’t marked difference of mRNA levels of SCAMP1, SCAMP3 and SCAMP4 between AML individuals and normal controls, the overall survival rates also showed negatively corelative with the expression of SCAMPs. In addition to SCAMP2 and SCAMP5, SCAMP4 mRNA level also showed significantly negative correlation with survival rate. Together, patients with higher expression levels of SCAMPs, especially SCAMP2, 4 and 5, showed lower survival rates, suggesting the potential role of SCAMPs as prognostic markers.

### Predicted functions and pathways related with SCAMP2/4/5 and their co-expressed genes in patients with AML

To explore the functional pathways involved in SCAMP2/4/5 mediated prognosis, we further performed co-expression analysis on SCAMP2/4/5 using the LinkedOmics Database. As shown in Table 2, the top 50 co-expressed genes of SCAMP2/4/5, respectively, in AML (LinkedOmics) were listed. The cell cycle-related genes such as *WAS, CTDSP1, G6PD* and *GPX1* were significantly co-expressed with SCAMP2; genes such as *ADAT3, CSNK1G2* and *BAT3* were significantly co-expressed with SCAMP4; and gene such as *KCTD5, BLNK* and *GUCY2D* were significantly co-expressed with SCAMP5.

**Table 2:** The top 50 co-expressed genes of SCAMPs in AML (LinkedOmics).

SCAMP2				SCAMP4				SCAMP5			
Positive		Negative		Positive		Negative		Positive		Negative	
Query	Statistic	Query	Statistic	Query	Statistic	Query	Statistic	Query	Statistic	Query	Statistic
*WAS*	0.70	*CEP290*	− 0.65	*ADAT3*	0.81	*GOLGA4*	− 0.63	*KCTD5*	0.43	*KCNQ5*	− 0.37
*CTDSP1*	0.68	*THUMPD1*	− 0.64	*CSNK1G2*	0.80	*VTA1*	− 0.63	*BLNK*	0.43	*DHX32*	− 0.35
*G6PD*	0.67	*PIBF1*	− 0.64	*BAT3*	0.75	*TROVE2*	− 0.62	*GUCY2D*	0.41	*ZADH2*	− 0.34
*GPX1*	0.67	*CCDC41*	− 0.62	*ZDHHC8*	0.74	*RBM41*	− 0.62	*ST6GALNAC4*	0.41	*SMARCA2*	− 0.33
*GNAI2*	0.67	*EPT1*	− 0.62	*REXO1*	0.74	*ZNF654*	− 0.62	*LEPREL1*	0.40	*PLD1*	− 0.33
*RAB5C*	0.67	*THOC2*	− 0.62	*C19orf6*	0.73	*HINT3*	− 0.62	*RPP25*	0.39	*ZNF35*	− 0.33
*RGS19*	0.66	*WRN*	− 0.62	*PTPN23*	0.73	*CWC22*	− 0.62	*IRF8*	0.38	*ZNF529*	− 0.33
*KIAA1949*	0.66	*UBA5*	− 0.59	*SFRS16*	0.73	*ESCO1*	− 0.61	*CYTH4*	0.38	*GLMN*	− 0.33
*GPSM3*	0.66	*AKD1*	− 0.59	*CIC*	0.72	*ANKRD12*	− 0.61	*OTOA*	0.37	*GPATCH2*	− 0.33
*CSK*	0.66	*AKAP9*	− 0.59	*NCLN*	0.72	*RAB5A*	− 0.61	*GAS6*	0.37	*REV1*	− 0.32
*RHOG*	0.66	*RBM26*	− 0.58	*C19orf29*	0.72	*PPP1R12A*	− 0.61	*MX1*	0.37	*UBA6*	− 0.32
*MGAT1*	0.65	*REV1*	− 0.58	*MGRN1*	0.72	*NEK7*	− 0.61	*CUEDC1*	0.37	*SPRYD4*	− 0.32
*CORO1A*	0.65	*OSBPL9*	− 0.58	*MIB2*	0.72	*SLMAP*	− 0.60	*IL28RA*	0.37	*ZNF233*	− 0.32
*SHKBP1*	0.65	*SUV39H2*	− 0.58	*CNOT3*	0.71	*CUL5*	− 0.60	*FUT7*	0.37	*DPY19L3*	− 0.32
*TMEM127*	0.65	*MTPAP*	− 0.58	*STRN4*	0.71	*SMEK2*	− 0.60	*LAT2*	0.36	*MYB*	− 0.32
*SH3BGRL3*	0.64	*RINT1*	− 0.58	*BTBD2*	0.71	*TXNDC9*	− 0.60	*TUBG2*	0.36	*GALNT1*	− 0.32
*TGFB1*	0.64	*LARP1B*	− 0.58	*COBRA1*	0.71	*MOBKL3*	− 0.60	*CDH23*	0.36	*MIB1*	− 0.31
*ABCD1*	0.64	*NCBP2*	− 0.58	*RNF31*	0.71	*RAB3GAP2*	− 0.60	*VAV1*	0.36	*KDM5B*	− 0.31
*MTMR14*	0.64	*PTCD3*	− 0.57	*MLL4*	0.71	*RBM43*	− 0.60	*C1R*	0.36	*EXT2*	− 0.31
*RNPEPL1*	0.64	*DLG1*	− 0.57	*HMG20B*	0.71	*DCUN1D1*	− 0.59	*LILRA4*	0.35	*PTPN14*	− 0.31
*ITPK1*	0.63	*C1orf27*	− 0.57	*ATP13A1*	0.71	*ARL5A*	− 0.59	*PDLIM3*	0.35	*ZC3H14*	− 0.31
*TFE3*	0.63	*FASTKD2*	− 0.57	*KLF16*	0.71	*SEC22B*	− 0.58	*NAPA*	0.35	*GBE1*	− 0.31
*FERMT3*	0.63	*CCDC52*	− 0.57	*ATXN2L*	0.70	*METT5D1*	− 0.58	*ZNF532*	0.35	*RPAP2*	− 0.30
*WBP2*	0.63	*TPP2*	− 0.57	*POLRMT*	0.70	*HAUS6*	− 0.58	*CLEC4C*	0.35	*CLINT1*	− 0.30
*MAP7D1*	0.63	*KRR1*	− 0.57	*NUBP2*	0.70	*TRIM33*	− 0.58	*TFEB*	0.35	*PIGV*	− 0.30
*ZYX*	0.63	*ZNF326*	− 0.57	*MED16*	0.70	*RPS6KB1*	− 0.58	*HERPUD1*	0.35	*ZDHHC21*	− 0.30
*EPN1*	0.63	*XPO4*	− 0.56	*TP53I13*	0.70	*ZNF148*	− 0.57	*SH3TC1*	0.35	*MTMR2*	− 0.30
*CAPNS1*	0.62	*THOC1*	− 0.56	*SGTA*	0.70	*ZNF267*	− 0.57	*SERPINF1*	0.35	*KIAA1958*	− 0.30
*ARRB2*	0.62	*SR140*	− 0.56	*FBRS*	0.70	*THUMPD3*	− 0.57	*ABLIM3*	0.35	*CSPP1*	− 0.30
*SPRYD3*	0.62	*TRNT1*	− 0.56	*C19orf28*	0.70	*SFRS2IP*	− 0.57	*RHOBTB2*	0.34	*COPB1*	− 0.30
*CORO7*	0.62	*SFRS13A*	− 0.56	*GIT1*	0.70	*CDC40*	− 0.57	*ATP13A2*	0.34	*ADAM17*	− 0.30
*AP2A1*	0.61	*MIB1*	− 0.56	*E4F1*	0.69	*C10orf78*	− 0.57	*LOC349114*	0.34	*SOCS6*	− 0.30
*SHISA5*	0.61	*GOLGB1*	− 0.56	*MBD3*	0.69	*CGGBP1*	− 0.57	*PNOC*	0.34	*WARS2*	− 0.30
*UNC93B1*	0.61	*CCDC76*	− 0.56	*SCAF1*	0.69	*SDCCAG1*	− 0.56	*C9orf142*	0.34	*FNDC3B*	− 0.29
*CAPN1*	0.61	*RRP15*	− 0.56	*INTS1*	0.69	*ERGIC2*	− 0.56	*SMTN*	0.34	*FANK1*	− 0.29
*C6orf1*	0.60	*CLUAP1*	− 0.56	*RNF126*	0.69	*ZNF37A*	− 0.56	*SH2D3C*	0.34	*SNX14*	− 0.29
*WDR1*	0.60	*PCM1*	− 0.56	*SOLH*	0.69	*API5*	− 0.56	*PLD4*	0.34	*LPO*	− 0.29
*CFL1*	0.60	*CHD9*	− 0.56	*TSC2*	0.69	*GNB4*	− 0.56	*C17orf28*	0.34	*SMAD5*	− 0.29
*GSK3A*	0.60	*DHX36*	− 0.56	*RPUSD1*	0.68	*THAP5*	− 0.56	*SMOC1*	0.34	*TRNT1*	− 0.29
*PPP1R9B*	0.60	*CCAR1*	− 0.56	*HGS*	0.68	*CPSF2*	− 0.56	*METTL11A*	0.34	*CASP6*	− 0.29
*TWF2*	0.60	*PRPF4B*	− 0.56	*SF3A2*	0.68	*RPAP3*	− 0.56	*WNT5A*	0.34	*PWP1*	− 0.29
*EFHD2*	0.59	*C10orf4*	− 0.56	*RAB11B*	0.68	*EEA1*	− 0.56	*PPCDC*	0.34	*TMEM232*	− 0.29
*MCOLN1*	0.59	*FAM179B*	− 0.56	*SYVN1*	0.68	*YME1L1*	− 0.56	*DPPA3*	0.34	*PRELID2*	− 0.29
*TICAM1*	0.59	*RPAP2*	− 0.56	*SGSM3*	0.68	*FAM126B*	− 0.56	*FMO6P*	0.33	*ANXA8L2*	− 0.29
*MKL1*	0.59	*CCDC138*	− 0.56	*USF2*	0.68	*XIAP*	− 0.56	*SEMA4D*	0.33	*ATP2C1*	− 0.29
*RAC2*	0.59	*MBTD1*	− 0.56	*C9orf86*	0.68	*ATF1*	− 0.56	*NAPSB*	0.33	*LOC647979*	− 0.29
*ATP6V0C*	0.59	*CSPP1*	− 0.55	*EDC4*	0.68	*LIN7C*	− 0.56	*TACC3*	0.33	*GTF3C3*	− 0.29
*IRF5*	0.59	*ESF1*	− 0.55	*VPS4A*	0.67	*LRRC40*	− 0.56	*CDC25B*	0.33	*FAM20B*	− 0.28
*CAPZB*	0.59	*HNRNPH3*	− 0.55	*SLC25A22*	0.67	*KIAA0776*	− 0.56	*SCNN1B*	0.33	*ZFP112*	− 0.28
*VASP*	0.59	*ITGB3BP*	− 0.55	*CRTC2*	0.67	*ORC2L*	− 0.56	*FAM78A*	0.33	*BCL2L2*	− 0.28

**Figure 4:**
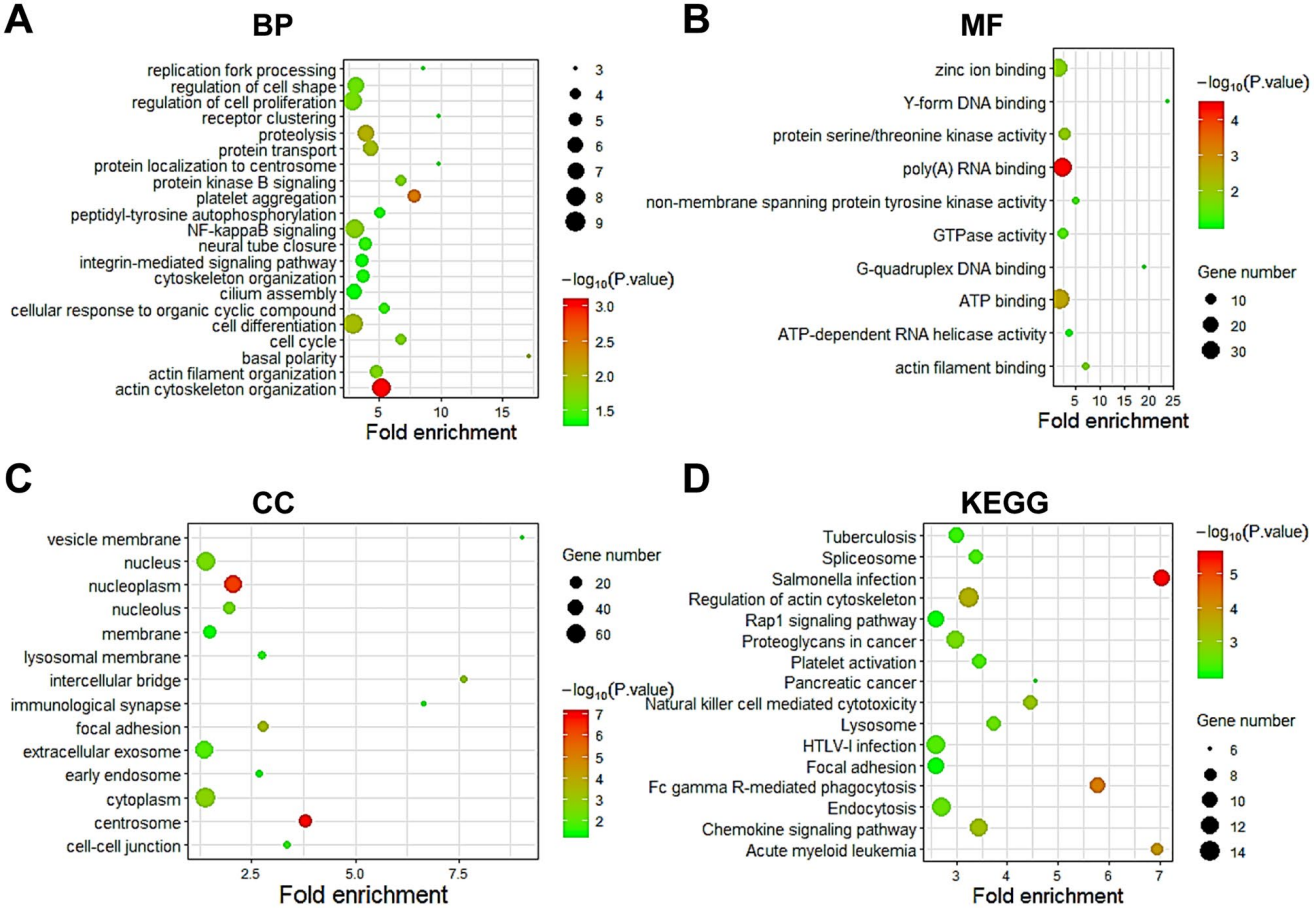
GO and KEGG enrichment analyses of SCAMP2 in AML (DAVID database). (**A**) Biological processes (BPs), (**B**) Molecular functions (MFs), (**C**) Cellular components (CCs) and (**D**) KEGG pathway related to the function of genes.

**Figure 5:**
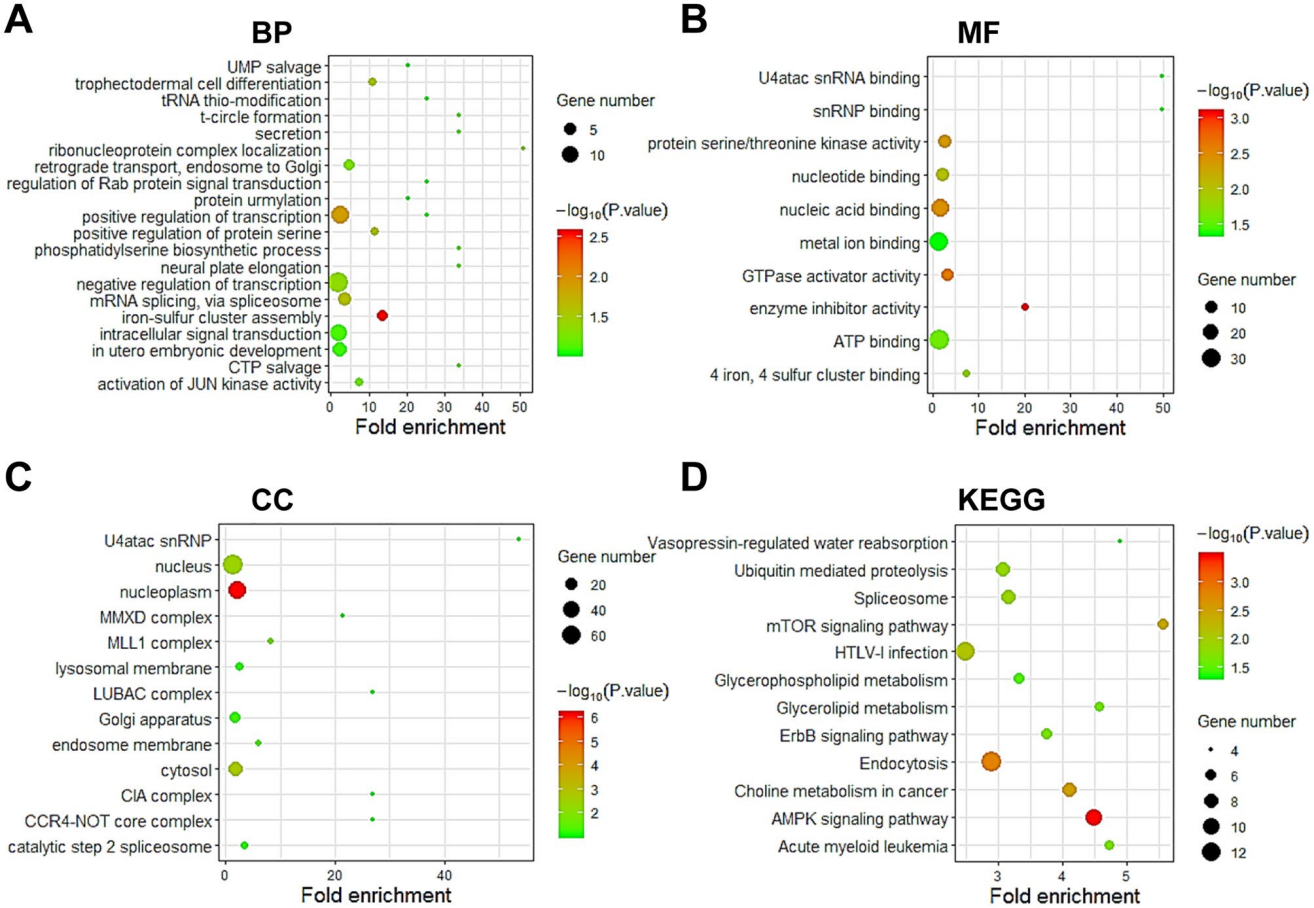
GO and KEGG enrichment analysis of SCAMP4 in AML (DAVID database). (**A**) Biological processes (BPs), (**B**) Molecular functions (MFs), (**C**) Cellular components (CCs) and (**D**) KEGG pathway related to the function of genes.

**Figure 6:**
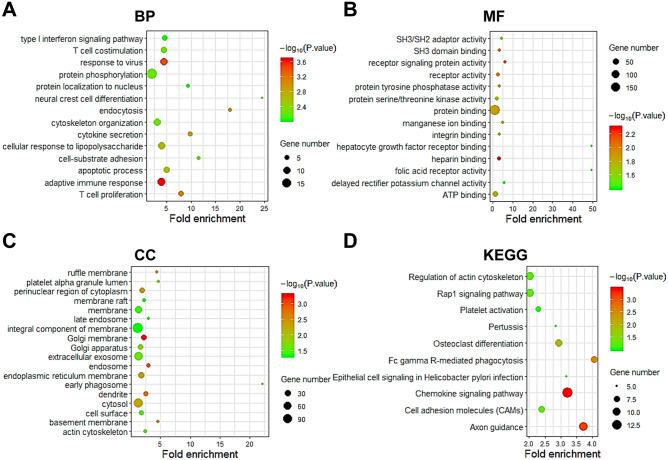
GO and KEGG enrichment analysis of SCAMP5 in AML (DAVID database). (**A**) Biological processes (BPs), (**B**) Molecular functions (MFs), (**C**) Cellular components (CCs) and (**D**) KEGG pathway related to the function of genes.

We employed GO and KEGG combining with R in the DAVID to predict the function and signal pathways of SCAMP2/4/5 and their co-expressed genes (Figs. 4, 5, and 6, Tables 3  and  4). For SCAMP2, the related biological processes (BP) such as cell proliferation, cell differentiation and cell cycle, the molecular functions (MF) such as protein serine/threonine kinase activity and poly (A) RNA binding, the cellular components (CC) such as cytoplasm, focal adhesion and cell–cell junction (Fig. 4A–C, Table 3), and the KEGG pathways such as Rap-1 signaling pathway, Fc gamma R-mediated phagocytosis, and AML signaling pathway (Fig. 4D, Table 4) were remarkably regulated by the SCAMP2 co-expressed genes.

**Table 3:** The top 5 GO function enrichment analysis of SCAMPs and co-expressed genes in AML (DAVID).

Category	Term	Description	Count	*P*. Value
**SCAMP2**				
BP	GO:0,030,154	Cell differentiation	9	1.14E-02
BP	GO:0,030,036	Actin cytoskeleton organization	8	8.64E-04
BP	GO:0,007,015	Actin filament organization	5	2.01E-02
BP	GO:0,007,049	Cell cycle	4	2.02E-02
BP	GO:0,045,197	Basal polarity	3	1.21E-02
MF	GO:0,044,822	Poly(A) RNA binding	35	3.68E-05
MF	GO:0,051,015	Actin filament binding	4	1.79E-02
MF	GO:0,005,524	ATP binding	39	2.41E-03
MF	GO:0,004,004	ATP-dependent RNA helicase activity	4	9.71E-02
MF	GO:0,051,880	G-quadruplex DNA binding	2	9.95E-02
CC	GO:0,005,737	Cytoplasm	74	1.83E-03
CC	GO:0,005,634	Nucleus	68	3.66E-03
CC	GO:0,070,062	Extracellular exosome	59	1.11E-02
CC	GO:0,005,654	Nucleoplasm	54	4.55E-07
CC	GO:0,016,020	Membrane	26	4.05E-02
**SCAMP4**				
BP	GO:0,000,122	Negative regulation of transcription	14	4.26E-02
BP	GO:0,045,893	Positive regulation of transcription	11	1.29E-02
BP	GO:0,000,398	mRNA splicing, via spliceosome	6	2.44E-02
BP	GO:0,016,226	Iron-sulfur cluster assembly	4	2.83E-03
BP	GO:0,071,902	Positive regulation of protein serine	3	2.58E-02
MF	GO:0,005,524	ATP binding	34	2.66E-02
MF	GO:0,046,872	Metal ion binding	30	4.38E-02
MF	GO:0,003,676	Nucleic acid binding	26	3.61E-03
MF	GO:0,000,166	Nucleotide binding	13	9.76E-03
MF	GO:0,004,674	Protein serine/threonine kinase activity	12	4.38E-03
CC	GO:0,005,634	Nucleus	67	3.75E-03
CC	GO:0,005,654	Nucleoplasm	50	7.07E-07
CC	GO:0,005,829	Cytosol	30	1.89E-03
CC	GO:0,005,794	Golgi apparatus	15	5.60E-02
CC	GO:0,071,339	MLL1 complex	4	1.20E-02
**SCAMP5**				
BP	GO:0,006,468	Protein phosphorylation	19	5.60E-03
BP	GO:0,006,955	Immune response	17	1.22E-02
BP	GO:0,002,250	Adaptive immune response	12	2.16E-04
BP	GO:0,009,615	Response to virus	10	3.97E-04
BP	GO:0,007,010	Cytoskeleton organization	10	5.58E-03
MF	GO:0,005,515	Protein binding	198	3.57E-01
MF	GO:0,005,524	ATP binding	43	7.75E-02
MF	GO:0,004,872	Receptor activity	11	1.98E-02
MF	GO:0,008,201	Heparin binding	10	1.80E-02
MF	GO:0,017,124	SH3 domain binding	8	1.44E-02
CC	GO:0,005,829	Cytosol	84	5.64E-03
CC	GO:0,005,789	Endoplasmic reticulum membrane	29	4.98E-03
CC	GO:0,000,139	Golgi membrane	25	5.22E-04
CC	GO:0,005,794	Golgi apparatus	27	1.64E-02
CC	GO:0,048,471	Perinuclear region of cytoplasm	23	4.67E-03

**Table 4:** The top 5 KEGG function enrichment analysis of SCAMPs and co-expressed genes in AML (DAVID).

Category	Term	Description	Count	*P*. Value	FDR
**SCAMP2**					
KEGG	hsa04062	Chemokine signaling pathway	14	3.68E-04	0.47
KEGG	hsa04360	Axon guidance	11	7.07E-04	0.90
KEGG	hsa04666	Fc gamma R-mediated phagocytosis	9	1.11E-02	13.25
KEGG	hsa04380	Osteoclast differentiation	8	3.26E-03	4.08
KEGG	hsa04514	Cell adhesion molecules (CAMs)	8	4.72E-02	46.00
**SCAMP4**					
KEGG	hsa04144	Endocytosis	13	1.55E-03	1.88
KEGG	hsa05166	HTLV-I infection	12	8.05E-03	9.40
KEGG	hsa04152	AMPK signaling pathway	10	3.45E-04	0.42
KEGG	hsa05231	Choline metabolism in cancer	8	3.12E-03	3.75
KEGG	hsa03040	Spliceosome	8	1.25E-02	14.30
**SCAMP5**					
KEGG	hsa04062	Chemokine signaling pathway	14	3.68E-04	0.47
KEGG	hsa04360	Axon guidance	11	7.07E-04	0.90
KEGG	hsa04380	Osteoclast differentiation	9	1.11E-02	13.25
KEGG	hsa04666	Fc gamma R-mediated phagocytosis	8	3.26E-03	4.08
KEGG	hsa04514	Cell adhesion molecules (CAMs)	8	4.72E-02	46.00

Similarly, for SCAMP4 and its co-expressed genes, the BPs were concentrated in process related to the regulation of transcription and mRNA splicing via spliceosome. The MFs for these genes included transcriptional regulation by ATP binding and protein serine/threonine kinase activity. These genes were associated with CCs including the nucleus and MLL1 complex (Fig. 5A–C, Table 3). Additionally, the KEGG analysis revealed significant enrichment of genes in endocytosis, AMPK, choline metabolism in cancer and spliceosome signaling pathways (Fig. 5D, Table 4).

Finally, for SCAMP5 and its co-expressed genes, several BPs were involved, including the regulation of protein phosphorylation, immune response, adaptive immune response and cytoskeleton organization. The MFs, including protein binding, ATP binding, receptor activity, heparin binding and SH3 domain binding, were affected by these genes. CCs, including cytosol, endoplasmic reticulum membrane and perinuclear region of cytoplasm, were significantly associated with these genes (Fig. 6C, Table 3). Additionally, there were multiple KEGG pathways for the SCAMP5 and its co-expressed genes, including chemokine, axon guidance, Fc gamma R-mediated phagocytosis and cell adhesion molecule signaling pathways (Fig. 6D, Table 4).

### Functional enrichment analysis of genes co-expressing with SCAMP2/4/5 in AML

**Figure 7:**
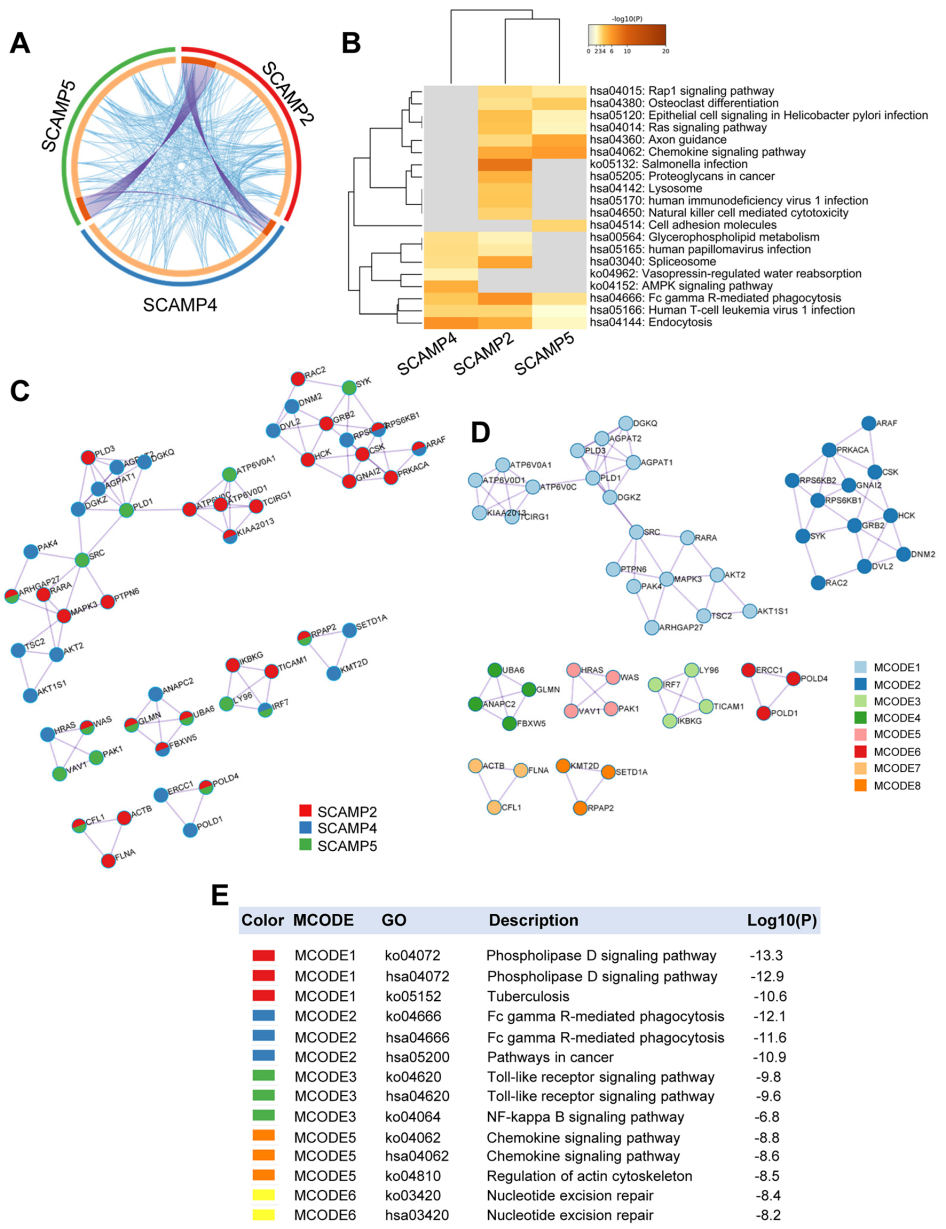
Overlaps, enrichment analysis, PPI network and MCODE analysis of SCAMP2/4/5 and their co-expressed genes in Metascape (Metascape database). (**A**) Circus plot of overlaps among SCAMP2/4/5 and their co-expressed genes. (**B**) Heatmap of enriched terms among SCAMP2/4/5 and their co-expressed genes. (**C**) Protein–protein interaction (PPI) network among SCAMP2/4/5 and their co-expressed genes. (**D**) MCODE components were identified in PPI network among SCAMP2/4/5 and their co-expressed genes. (**E**) Five MCODE components list in PPI network among SCAMP2/4/5 and their co-expressed genes.

To explore the interactions and internal mechanisms of co-expressed genes of SCAMP2/4/5, we used Metascape to perform the overlap analysis, enrichment analysis, PPI network and MCODE analysis of SCAMP2/4/5 and their co-expressed genes. We identified specific overlap genes among SCAMP2/4/5 and their co-expressed genes (Fig. 7A). The top 20 KEGG pathways for the genes co-expressing with SCAMP2/4/5 are shown in Fig. 7B and Table 5. What is worth mentioning is that the gene set was responsible for the AML pathway. In addition, the gene set was also involved in chemokine signaling pathway, axon guidance and cell adhesion molecule signaling pathways. To better understand the potential biological mechanisms between SCAMP2/4/5 and leukemia, we used Metascape to generate the PPI network of the gene set (Fig. 7C), and found several significant MCODE components from the PPI network according to the clustering scores (Fig. 7D). Importantly, enrichment analysis applied to each MCODE component indicated that biological function was primarily associated with series of pathways in cancer and immunity (Fig. 7E).

**Table 5:** Top 20 clusters with meta-analysis of KEGG enrichment pathway of SCAMP2/4/5 and their co-expressed genes.

GO	Description	Count	%	Log10(P)	Log10(q)
hsa04666	Fc gamma R-mediated phagocytosis	22	1.96	− 9.75	− 6.92
hsa04144	Endocytosis	36	3.20	− 9.53	− 6.92
Ko05132	Salmonella infection	12	3.01	− 8.48	− 5.57
hsa05166	Human T-cell leukemia virus 1 infection	34	3.02	− 7.45	− 5.25
hsa04062	Chemokine signaling pathway	26	2.31	− 6.79	− 4.79
hsa03040	Spliceosome	20	1.78	− 6.41	− 4.58
hsa04014	Ras signaling pathway	29	2.58	− 6.19	− 4.41
hsa00564	Glycerophospholipid metabolism	15	1.34	− 4.5	− 2.66
hsa05165	Human papillomavirus infection	35	3.11	− 6.09	− 4.39
hsa04360	Axon guidance	24	2.13	− 5.93	− 4.25
hsa05205	Proteoglycans in cancer	25	2.22	− 5.62	− 3.99
hsa05120	Epithelial cell signaling in Helicobacter pylori infection	13	1.16	− 5.38	− 3.78
ko04152	AMPK signaling pathway	10	2.51	− 5.08	− 3.45
hsa04380	Osteoclast differentiation	18	1.60	− 4.98	− 3.54
hsa04142	Lysosome	17	1.51	− 4.54	− 3.18
hsa04015	Rap1 signaling pathway	23	2.04	− 4.32	− 3.01
hsa05170	human immunodeficiency virus 1 infection	12	3.01	− 3.94	− 2.62
hsa04650	Natural killer cell mediated cytotoxicity	9	2.26	− 3.56	− 2.33
ko04962	Vasopressin-regulated water reabsorption	8	0.71	− 3.50	− 2.34
hsa04514	Cell adhesion molecules	9	2.27	− 3.47	− 2.30

## Discussion

In this study, we collected several sets of data from multiple databases including Oncomine, GEPIA, cBioPortal, LinkedOmics and DAVID, and performed comprehensive bioinformatic analysis to evaluate the role of SCAMPs in AML. We did not find significant correlation between SCAMPs gene mutation and AML prognosis. However, the SCAMP2/5 mRNA levels were significantly higher in AML patients than in healthy controls. Moreover, high mRNA expressions of SCAMP2/4/5 were associated with poor overall survival. We further found that SCAMP2/4/5 and their co-expressed genes were associated with multiple pathways related to tumorigenesis and progression.

Though the fact that there is a relationship between SCAMPs and multiple human cancers, the function of SCAMPs from cancer to cancer varies. For example, Vadakekolathu et al. found that SCAMP1 facilitates MTSS1 (metastasis suppressor protein 1) transport to cell surface and cooperate to prevent HER2 + /ER-/PR- breast cancer invasion, indicating SCAMP1 as a tumor repressor in breast cancer^5^. On the contrast, SCAMP1 also showed tumor-promoting activity in other solid cancer. Zong et al. found that highly-expressed SCAMP1 in glioma facilitated malignant progression and suppressed apoptosis of glioma cells by regulating the miR-499a-5p/LMX1A/NLRC5 pathway, which was associated with poor overall survival^32^. SCAMP1 has also identified as a lymph node metastasis-associated marker in pancreatic and gallbladder cancers^6^ and loss of SCAMP1 has also shown to improve overall survival in pancreatic adenocarcinoma^13^. These arguments may result from tumor heterogeneity. To date, specific roles of SCAMP members in AML are yet obscure. This study conducted a comprehensive assessment of the prognostic relevance of SCAMPs in AML via multiple bioinformatics analysis and suggest that SCAMP2/5 are potential diagnostic markers for AML, and SCAMP2/4/5 are potential prognostic markers for AML.

We found that SCAMP1 expression levels showed increased in AML patients compared to normal controls in Andersson Datasets, which is consistent with a previous report^4^. However, in the GEPIA database, SCAMP1 also showed a trend of increased expression in AML patients compared to normal controls, but without significant difference. Thus, the diagnostic role of SCAMP1 needs to be further clarified.

SCAMP3, an important membrane carrier, was reported to participate in cell growth by interacting with DRAM-1, which was in turn involved in the activation of mTORC1^33,34^. Overexpression of SCAMP3 was found to be closely related to poor overall survival in human hepatocellular carcinoma and that knockdown of SCAMP3 decreased cell proliferation and cell cycle progression of HCC cells^11^. This suggested that SCAMP3 may play a carcinogenic role. However, SCAMP3 was also reported to function as a novel tumor suppressor in lung cancer by modulating EGFR signaling and cytokinesis^12^. Herein, the expression of SCAMP3 in AML tissues was found higher than normal controls from Oncomine database. Despite there being no correlation between expression of SCAMP3 and prognosis of AML in our study, SCAMP3 may play an important role in regulating AML.

Previous research has reported that overexpression of SCAMP4 promoted the secretion of senescence-associated secretory phenotype (SASP) factors and also affected cell proliferation of WI-38 cells expressing SCAMP4-Myc, in contrast to cells only expressing the Myc tag^35^. However, SASP was a major trait of senescent cells^36^. In addition, an in vitro study demonstrated that senescent fibroblasts overexpressing SASP may potentially stimulate or accelerate neoplastic progression by creating a tumorigenic microenvironment^37^. Similarly, the growth of leukemia cells created abnormal bone marrow microenvironments, which played a key role in the initiation and development of hematological malignancies^38^. This raised the question if SASP plays the same role in leukemia. In our report, we found that a higher SCAMP4 expression significantly correlated with poor overall survival, pointing to SCAMP4 as a possible tumor promoter. The molecular mechanism involved in SCAMP4 in AML and whether elevated SCAMP4 in AML promotes the secretion of SASP is the next step that needs to be investigated.

Until now, little was known about the expressions and specific roles of SCAMP2 and SCAMP5 in AML. SCAMP2 was shown to set up soluble N-ethylmaleimide sensitive factor attachment protein receptors (SNAREs) interactions and has an essential function in granule exocytosis by fusion pore formation^39–41^. Similarly, SCAMP5 is directly involved in calcium-regulated exocytosis of signal peptide-containing cytokines via co-distributing and compelling with SNAREs^10^. Interestingly, increasing evidence has proven that SNAREs played a core role in vesicle transport by vesicle-target membrane fusion, which was vital for compartment integrity, exocytosis and trafficking within the cell^42–47^. In addition, SNAREs play an essential role in the delivery of mutant KRAS from recycling endosome to plasma membrane through vesicular transport, which facilitated KRAS associating with downstream effectors to carry out its tumorigenic action^47^. Nevertheless, whether the docking sites of SNARE and SCAMP2/5 are involved in regulating other important signal transmission remained obscure. Herein, SCAMP2 and SCAMP5 were found significantly overexpressed in AML, and their expression showed positive correlation with each other. Importantly, high SCAMP2 and SCAMP5 expression was significantly associated with poor overall survival in AML patients, indicating the oncogenic roles of these transcriptional factors in AML. Further experimentation involving the docking sites of SNAREs and SCAMP2/5 may uncover the molecular mechanism of AML.

We constructed a network of SCAMP2/4/5 and 50 closest co-expressed genes for each of them. The results of the functional analysis of distinct SCAMP and its co-expressed genes indicated that these genes were involved in multiple pathways related to tumorigenesis and progression, such as human T-cell leukemia virus 1 (HTLV-1) infection, acute myeloid leukemia, and mTOR signaling pathways. The mTOR, downstream effector of PI3k, can make leukaemia-initiating cells acquire the properties of proliferation and survival and eliminate haematopoietic stem cell^48–50^. In AML, the activation of this pathway, can bring adversely prognostic impact to AML patients^51–53^.Taken together, these data suggest SCAMP2/4/5 may be potential prognostic biomarkers for AML.

We also performed functional enrichment analysis of overlaps of SCAMP2/4/5 and their co-expressed genes in Metascape to further prove the results of functional enrichment analysis above and explore the potential interaction mechanisms of SCAMP2/4/5 in AML. One of the interesting gene set was involved in NF-kappa B signaling pathway. In addition, NF-κB pathway, which was proved to be strongly correlated with TNF-α signaling, accounts for the progression of AML^54^. Our data including the potential mechanism involving NF-κB pathway suggest a vital role of SCAMP2/4/5 in tumorigenesis and progression of AML.

*In summary*, our results suggest that SCAMP2/4/5 are potential prognostic markers for AML, and that SCAMP2 and SCAMP5 individually or in combination may be used as diagnostic markers for AML.
